# The role of transcranial Doppler in predicting the incidence and prognosis of sepsis-associated encephalopathy

**DOI:** 10.1186/s40635-025-00826-9

**Published:** 2025-12-15

**Authors:** Hanady Mohammed Elfeky, Mohamed Basyouni Helal, Reham Naser Sherif, Sarah A. Nada, Walaa Samy Mokhtar, Hatem Amin AttaAllah, Yasser Ibrahim Fathy, Hala Mohamed Koptan

**Affiliations:** 1 Critical Care Medicine Department, Faculty of Medicine, Menoufia University, Shibin el Kom, 35211 Menoufia Egypt; 2 Faculty of Medicine, Menoufia University, Shibin el Kom, Menoufia Egypt; 3 Anesthesia, Intensive Care, and Pain Management Department, Faculty of Medicine, Menoufia University, Shibin el Kom, Menoufia Egypt

**Keywords:** Sepsis-associated encephalopathy, Transcranial Doppler, Pulsatility index, Resistive index, Sepsis

## Abstract

**Background:**

Sepsis-associated encephalopathy (SAE) is a common complication of sepsis, contributing to poor outcomes and increased mortality. Early detection remains challenging due to the absence of observable direct brain injury. Transcranial Doppler (TCD) ultrasonography provides a non-invasive, bedside tool for assessing cerebral hemodynamics and may help identify patients at risk. SAE was defined as new-onset delirium (positive CAM-ICU) or unexplained coma (GCS < 8) not attributable to structural or metabolic causes. This study aimed to evaluate the role of TCD in predicting the incidence and prognosis of SAE in septic patients admitted to the ICU.

**Methods:**

This prospective cohort study included 93 patients with sepsis. Demographic, clinical, and laboratory data were recorded upon admission. Daily TCD was performed for seven consecutive days to measure the pulsatility index (PI) and resistive index (RI). Neurological dysfunction was assessed daily using the Confusion Assessment Method for the ICU (CAM-ICU) and the Glasgow Coma Scale (GCS).

**Results:**

A total of 93 patients were included, of whom 44 (47.3%) developed SAE. SAE patients showed greater illness severity, with higher median SOFA (6 [5–7] vs. 5 [3–6], p < 0.001) and APACHE II (14 [12–17] vs. 11 [9–14], p < 0.001) scores, and higher 28-day mortality (61.4% vs. 22.4%, p < 0.001). The median PI and RI were consistently higher in SAE patients across all study days, while the mean flow velocity (mFV) was lower. PI on day 1 had the best accuracy for predicting SAE, with a cutoff ≥ 1.30 (AUC: 0.963, sensitivity 95.45%, specificity 100%). RI on day 3 was also highly predictive (AUC: 0.971, cutoff ≥ 0.67, sensitivity 95.45%, specificity 95.92%).

**Conclusions:**

In this sample of septic patients, PI and RI are strong predictors of SAE, with PI serving as a reliable early indicator of both SAE and mortality.

*Trial Preregistration* The study was registered in the Pan African Clinical Trials Registry: PACTR202410707982429, date: 7/10/2024.

**Supplementary Information:**

The online version contains supplementary material available at 10.1186/s40635-025-00826-9.

## Background

Sepsis-associated encephalopathy (SAE) is a serious complication of sepsis. It is a multifactorial condition marked by widespread brain dysfunction in response to uncontrolled systemic inflammation rather than direct brain injury. [1]. The pathogenesis of SAE is complex and primarily linked to cerebral hemodynamic abnormalities, including altered vasomotor tone and the release of inflammatory mediators, which disrupt cerebral blood flow and directly impair brain function [2]. Despite its clinical significance, SAE remains an underrecognized contributor to morbidity and mortality in septic patients [3, 4].

Given its strong association with altered consciousness and its frequent underdiagnosis as a cause of delirium in the intensive care unit (ICU), early identification and risk stratification of SAE are crucial. Prompt recognition can help optimize management strategies and improve patient prognosis. Various diagnostic modalities are available to assess brain dysfunction in primary brain injury, including radiological imaging. However, in SAE, radiological findings are often absent. Assessing cerebral blood flow abnormalities through conventional methods can be invasive and costly.

In this context, transcranial Doppler (TCD) serves as a non-invasive, real-time bedside technique for evaluating cerebral blood flow from the basal intracerebral vessels, providing rapid information and continuous monitoring of cerebral circulation [5]. It provides valuable insights into cerebral autoregulation, vasomotor reactivity, and perfusion changes that are significantly altered in sepsis [6]. Studies have shown that septic patients often exhibit impaired cerebral autoregulation, increased pulsatility index (PI), and changes in flow velocity patterns, all of which may serve as early indicators of impending neurological dysfunction [6].

The role of TCD in SAE prediction and prognosis is gaining increasing attention. By detecting early alterations in cerebral hemodynamics, TCD may serve as a valuable tool for identifying patients at high risk of developing SAE and predicting neurological outcomes [7]. Despite its potential, the clinical utility of TCD in SAE remains insufficiently studied, and further research is needed to establish standardized protocols, validate predictive parameters, and integrate TCD findings into routine sepsis care. Therefore, this study aimed to investigate the relationship between cerebral TCD findings and the development of SAE in patients with early septic shock. The primary outcome was the correlation between TCD parameters and the presence of SAE. Secondary outcomes included the association of TCD findings with ICU length of stay and in-hospital mortality.

## Methods

### Study design

This prospective cohort study was conducted in the critical care units of Menoufia University Hospitals. Ethical approval was obtained from the Institutional Review Board (IRB) of Menoufia University, Menoufia, Egypt (Approval No. 7/2023 ANET). Informed consent was obtained from all patients or their legal surrogates before enrollment. All adult patients with sepsis admitted to the ICU were screened for eligibility. The study enrolled 93 patients with early septic shock who underwent daily TCD examinations for seven consecutive days following sepsis diagnosis. Patients were monitored for the development of SAE and were subsequently classified into encephalopathy and non-encephalopathy groups.

### Sample size

Prior research has reported an area under the receiver operating characteristic (ROC) curve (AUC) of 0.73 (p = 0.0001) for the diagnostic performance of the resistive index (RI) as a predictor of subsequent sepsis-associated encephalopathy [8]. The sample size was calculated to be 93 subjects, with a study power of 80% and a confidence level of 95%.

We acknowledge that the study includes additional indices (e.g., Pulsatility Index (PI)) and evaluates data across multiple time points (Days 1 through 7). These were not included in the initial power estimation and are considered exploratory analyses.

### Study population

This study included adult patients aged 18 to 80 years who were admitted to the ICU with a diagnosis of sepsis or septic shock, as defined by the Third International Consensus Definitions for Sepsis and Septic Shock (Sepsis-3) [9]. Patients were eligible if the diagnosis had been made within the preceding 24 h of ICU admission or if sepsis developed during their ICU stay, provided enrollment occurred within 24 h of sepsis onset. Sepsis was defined as life-threatening organ dysfunction caused by a dysregulated host response to infection, identified by an acute increase of ≥ 2 points in the Sequential Organ Failure Assessment (SOFA) score [10]. Septic shock was defined as sepsis with persistent hypotension requiring vasopressor support to maintain a mean arterial pressure (MAP) ≥ 65 mmHg and a serum lactate level > 2 mmol/L, despite adequate fluid resuscitation.

To ensure reliable neurological assessment during the 7-day follow-up period, only patients with a baseline Glasgow Coma Scale (GCS) score of ≥ 13 (in the absence of sedation) and a negative CAM-ICU score were included, enabling detection of any degree of sepsis-associated encephalopathy. Mechanically ventilated patients receiving sedation were eligible if they were under light or conscious sedation at baseline, defined as a Richmond Agitation-Sedation Scale (RASS) score > − 1. This threshold was applied to ensure valid cognitive evaluation using the Confusion Assessment Method for the ICU (CAM-ICU) and GCS [11]. Additionally, patients were required to have normal arterial partial pressure of carbon dioxide (PaCO₂) levels (35–45 mmHg) to exclude confounding effects of hypercapnia or hypocapnia on mental status.

Exclusion criteria included death or ICU discharge before completion of the 7-day follow-up, pregnancy, and abnormal neuroimaging findings. Patients with known metabolic encephalopathies (e.g., uremic, hepatic, or hypoglycemic), significant electrolyte abnormalities (e.g., hyponatremia, hypernatremia, or hypokalemia), abnormal PaCO₂ levels, central nervous system infections (e.g., meningitis or encephalitis), or severe carotid artery stenosis (> 70%) were excluded. Other exclusions included alcohol- or drug-induced altered consciousness, pre-existing psychiatric or neurodegenerative disorders (e.g., Alzheimer’s disease or other dementias), and patients under deep sedation (RASS ≤ –2). Importantly, patients under deep sedation were excluded, not to deliberately omit more severe cases of SAE, but because accurate cognitive assessment using CAM-ICU was not feasible in this population. As the study did not incorporate neurophysiological tools such as electroencephalography (EEG), exclusion was necessary to ensure the reliability of clinical evaluations. We acknowledge that this approach may have led to an underrepresentation of patients with more profound SAE.

All patients received standard-of-care treatment for sepsis according to international guidelines, including prompt implementation of the first-hour sepsis bundle, hemodynamic optimization, and appropriate source control interventions.

### Study methods

All enrolled patients underwent TCD ultrasonography to evaluate cerebral hemodynamics. The initial TCD assessment was performed on the 1st day of ICU admission, after fluid resuscitation and MAP optimization, with or without vasopressor support. Serial TCD evaluations were conducted daily for seven days to assess early alterations in cerebral microcirculation potentially contributing to SAE.

During the 7-day follow-up period, we prospectively identified new-onset brain dysfunction using two clinical pathways: Delirium, as detected by a positive CAM-ICU score, and unexplained depressed consciousness, in the form of new-onset coma not attributable to structural brain lesions or metabolic disturbances (confirmed by normal brain CT and exclusion of other causes). All such cases were collectively categorized as SAE [12].

A diagnosis of SAE was made when patients met either of the following criteria:

- (1) Delirium as defined by CAM-ICU, which includes an acute onset or fluctuating course of mental status, inattention, and either an altered level of consciousness or disorganized thinking; or.
- (2) Unexplained depressed consciousness, manifested as new-onset coma (GCS < 8), not attributable to structural brain lesions or metabolic disturbances.

For patients requiring mechanical ventilation, a baseline CAM-ICU assessment was performed before intubation whenever feasible. Sedation levels were monitored using the RASS score. Only patients with a RASS score > − 1 were included to ensure sufficient arousability for cognitive testing. Patients under deep sedation (RASS ≤ –2) were excluded due to the inability to perform reliable neurological assessments. Conscious sedation was maintained using dexmedetomidine at a dose of 0.2–0.7 µg/kg/hour, titrated to achieve light sedation without causing respiratory depression, thereby enabling repeated neurological evaluations.

Patients were prospectively categorized into two groups based on the presence or absence of SAE. Primary outcomes included differences in cerebral hemodynamic parameters, particularly PI and RI. Secondary outcomes were ICU length of stay and mortality at 7 and 28 days.

Demographic and clinical data—including age, sex, weight, and comorbidities such as hypertension, diabetes mellitus, cardiac, renal, or hepatic disorders—were recorded at ICU admission. Vital parameters such as MAP, heart rate, oxygen saturation (SpO₂), and fluid balance were continuously monitored. Laboratory investigations included hemoglobin, leukocyte count, serum lactate, C-reactive protein (CRP), procalcitonin, creatinine, blood urea nitrogen (BUN), liver enzymes (ALT, AST), and serum electrolyte levels. Severity of illness was assessed using both the Acute Physiology and Chronic Health Evaluation II (APACHE II) score [13] and the SOFA score, measured at admission and on day seven.

Brain CT or MRI imaging was performed when clinically indicated to rule out cerebrovascular events.

### TCD measurements

TCD assessments were performed daily by a trained operator who was not involved in the clinical management of the patients. Importantly, the operator was blinded to the patients' status, as determined by the CAM-ICU assessments or GCS. This ensured that TCD data collection was conducted independently, minimizing observer bias.

A bedside ultrasound machine with a TCD preset was used, with the index marker positioned to the left of the ultrasound screen. A phased array probe (2–5 MHz) equipped with color Doppler and pulse-wave Doppler (PWD) modes was utilized for the examination. The middle cerebral arteries (MCAs) were insonated through the transtemporal window at a depth of 50–60 mm.

For the examination, patients were positioned supine while the operator stood on the patient’s right side. A small amount of ultrasound gel was applied to the temporal bone, just anterior to the ear, at the level of the eye. The probe’s index marker was directed anteriorly toward the patient’s eyes. The examination began with color Doppler to identify the MCA, followed by PWD placement parallel to blood flow over the artery with a sample volume of 4 mm to obtain velocity measurements.

Hemodynamic parameters recorded included the time-averaged mean velocity (FVm, cm/s), peak systolic velocity (FVs, cm/s), end-diastolic velocity (FVd, cm/s), PI, and RI. Both PI and RI were used to assess cerebrovascular resistance.

To ensure measurement reproducibility and reliability, each velocity measurement on both sides of the brain was repeated three times. The highest value from each side was considered, and the average of the right and left MCA values was used for analysis.

### Statistical analysis

Data was analyzed using IBM SPSS Statistics for Windows Version 29 (IBM Corp., Armonk, N.Y., USA). Categorical variables were summarized as frequencies and percentages. The normality of continuous variables was assessed using the Shapiro–Wilk test and the Kolmogorov–Smirnov test. Normally distributed data were summarized as mean ± standard deviation (SD), while non-normally distributed data were presented as median and interquartile range (IQR). Comparisons between groups were conducted using an independent samples t-test or Mann–Whitney U test for quantitative variables and the chi-square or Fisher's exact test for qualitative variables. Spearman’s rank correlation was applied to assess the correlation between PI, RI, and other continuous variables. Receiver Operating Characteristic (ROC) curve analysis was performed to evaluate the predictive performance of PI and RI. All analyses were performed on complete cases, and the number of observations included in each analysis is presented in the respective tables.

## Results

### Baseline characteristics and clinical outcomes

A total of 93 patients were included in the study, with 44 classified as SAE patients and 49 as non-SAE patients (Supplementary Fig. 1). SAE patients were significantly older than non-SAE patients (56 [50–58] vs. 50 [39–54] years, p < 0.001). However, there was no significant difference in the gender distribution (p = 0.490) or BMI between the two groups (p = 0.815). The prevalence of comorbidities—including diabetes mellitus, hypertension, chronic kidney disease, oncological disease, chronic liver disease, cardiac disease, obstructive lung disease, pulmonary embolism, and deep vein thrombosis—did not significantly differ between SAE and non-SAE patients.

Patients in the SAE group had significantly higher rates of mechanical ventilation (*p* = 0.004) and vasopressor use on Day 1 (*p* = 0.006) compared to the non-SAE group. By Day 7, vasopressor use was not significantly different (*p* = 0.652). Additionally, the SAE group had a significantly higher 28-day mortality rate (61.4% vs. 24.5%, *p* < 0.001). Among mechanically ventilated patients, the number of days of light sedation within the first 7 days was comparable between the two groups (5 [4, 5] vs. 4.5 [3.5–6] days, p = 0.932). Among patients who developed SAE, the median time to encephalopathy onset was 4 (3–5) days. Most cases of SAE occurred on day 3 (13 cases, 29.6%) and day 5 (10 cases, 22.7%). The length of ICU stay did not differ significantly between groups (p = 0.091). SAE patients had substantially higher SOFA scores on day 1 and day 7 (6 [5–7] vs. 5 [3–6] and 8 [5–10] vs. 5 [3–6], respectively; p < 0.001 for both), as well as higher APACHE II scores (14 [12–17] vs. 11 [9–14], p < 0.001) **(**Table 1**).**

**Table 1:** Baseline characteristics, comorbidities, sources of sepsis, scores, and outcomes of the studied groups (N = 93)

Variable	SAE patients (n = 44)	Non-SAE patients (n = 49)	P-value
Gender, n (%)			0.490
-Female	22 (50.0%)	21 (42.9%)	
-Male	22 (50.0%)	28 (57.1%)	
Age (years), median (IQR)	56 (50–58)	50 (39–54)	< 0.001*
BMI, n (%)	23 (22–26)	23 (22–24)	0.815
DM, n (%)	34 (77.3%)	31 (63.3%)	0.141
HTN, n (%)	31 (70.5%)	30 (61.2%)	0.350
CKD, n (%)	15 (34.1%)	16 (32.7%)	0.883
Oncological disease, n (%)	20 (45.5%)	23 (46.9%)	0.886
Compensated chronic liver disease, n (%)	18 (40.9%)	14 (28.6%)	0.211
Cardiac disease, n (%)	33 (75.0%)	32 (65.3%)	0.309
Obstructive lung disease, n (%)	17 (38.6%)	18 (36.7%)	0.850
History of Pulmonary Embolism (PE), n (%)	3 (6.8%)	5 (10.2%)	0.718
Incident PE, n (%)	2 (4.5%)	4 (8.2%)	0.680
History of DVT, n (%)	9 (20.5%)	13 (26.5%)	0.626
Incident DVT, n (%)	8 (18.2%)	10 (20.4%)	1.000
Pneumonia on admission, n (%)	33 (75.0%)	32 (65.3%)	0.309
Urosepsis, n (%)	27 (61.4%)	32 (65.3%)	0.693
Abdominal sepsis, n (%)	16 (36.4%)	15 (30.6%)	0.557
Septic shock with vasopressors on Day 1, n (%)	38 (86.4%)	30 (61.2%)	0.006*
Septic shock with vasopressors on Day 7, n (%)	24 (54.5%)	29 (59.2%)	0.652
Mechanical ventilation under light sedation, n (%)	18 (40.9%)	7 (14.3%)	0.004*
Days of light sedation used in mechanically ventilated patients within 7 days, median (IQR)	5 (4–5)	4.5 (3.5–6)	0.932
Time to SAE onset (days), median (IQR)	4 (3–5)	–	–
Onset of SAE			
– Day 1	4 (9.1%)	–	–
– Day 2	4 (9.1%)	–	–
– Day 3	13 (29.6%)	–	–
– Day 4	6 (13.6%)	–	–
– Day 5	10 (22.7%)	–	–
– Day 6	5 (11.4%)	–	–
– Day 7	2 (4.5%)	–	–
ICU length of stay (days), median (IQR)	20 (15–25)	16 (12–22)	0.091
Mortality (on Day 28), n (%)	27 (61.4%)	12 (24.5%)	< 0.001*
SOFA score on Day 1, median (IQR)	6 (5–7)	5 (3–6)	< 0.001*
SOFA score on Day 7, median (IQR)	8 (5–10)	5 (3–6)	< 0.001*
APACHE II score, median (IQR)	14 (12–17)	11 (9–14)	< 0.001*

*APACHE II* Acute Physiology and Chronic Health Evaluation II, *BMI* Body Mass Index, *CAM-ICU*, Confusion Assessment Method for the ICU; *CKD* Chronic Kidney Disease, *DM* Diabetes Mellitus, *DVT* Deep Vein Thrombosis, *HTN* Hypertension; *ICU*, Intensive Care Unit; *SAE* Sepsis-Associated Encephalopathy, *SOFA* Sequential Organ Failure Assessment

P values for chi-square, independent samples t-test, or Mann–Whitney U test, as appropriate, were reported. * Statistically significant at P < 0.05

Some patients had more than one source of infection; therefore, percentages may exceed 100%

### Hemodynamic and laboratory parameters

Regarding the hemodynamic and laboratory parameters, SAE patients had significantly lower MAP after resuscitation on day 1 **(**73 [70–75] vs. 79 [77–81] mmHg, p < 0.001**)**, higher temperature on day 7 **(**38.2 [37.8–38.7] vs. 37.5 [37.3–37.6]°C, p < 0.001**)**, and higher heart rates on admission **(**117 ± 13 vs. 112 ± 11 bpm, p = 0.030**)** and day 7 **(**105 ± 14 vs. 93 ± 16 bpm, p < 0.001**)**. Procalcitonin levels were significantly higher in SAE patients (6.00 [1.85–11.50] vs. 2.00 [1.50–2.50] ng/mL, p < 0.001). Urea levels were also higher in SAE patients (45 [39–72] vs. 40 [19–50] mg/dL, p = 0.019). (Supplementary Table 1).

**Fig. 1:**
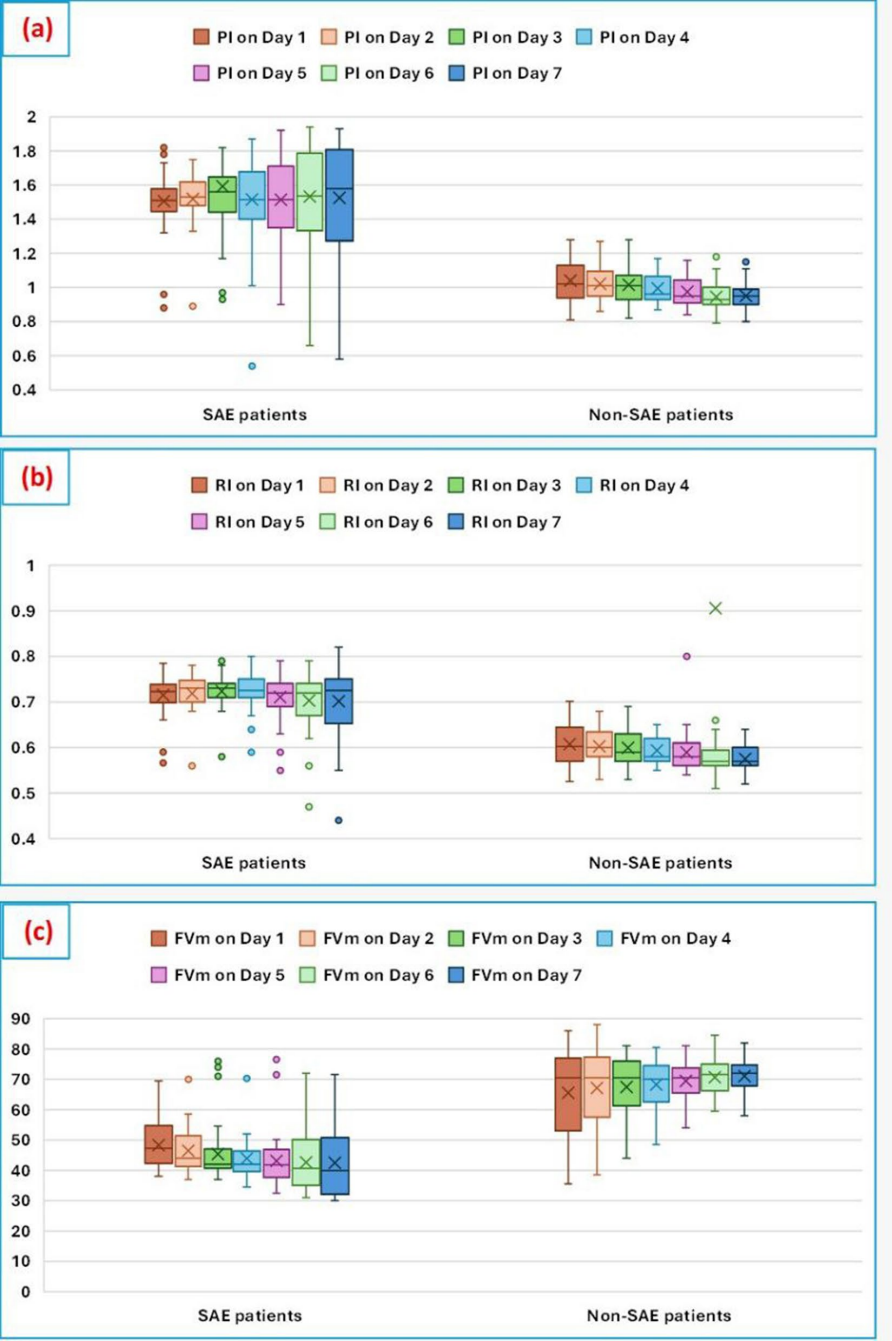
Box plots comparing (**a**) PI, (**b**) RI, and (**c**) FVm between SAE patients (n = 44) and non-SAE patients (n = 49) over 7 days (primary outcome). SAE patients consistently showed higher PI and RI and lower FVm values compared to non-SAE patients (all P < 0.001, Mann–Whitney U test). FVm, mean velocity; PI, Pulsatility Index; RI, Resistive Index.

### Cerebral hemodynamic indices

A comparison of cerebral hemodynamic parameters between SAE and non-SAE patients over seven days (Fig. 1) revealed that the pulsatility index (Fig. 1a) and resistance index (Fig. 1b) were significantly higher in SAE patients throughout the entire period (p < 0.001), whereas the mean velocity (Fig. 1c) was significantly lower in the SAE group (p < 0.001) (Supplementary Table 2).

### ROC analysis of the PI and RI for predicting SAE

**Fig. 2:**
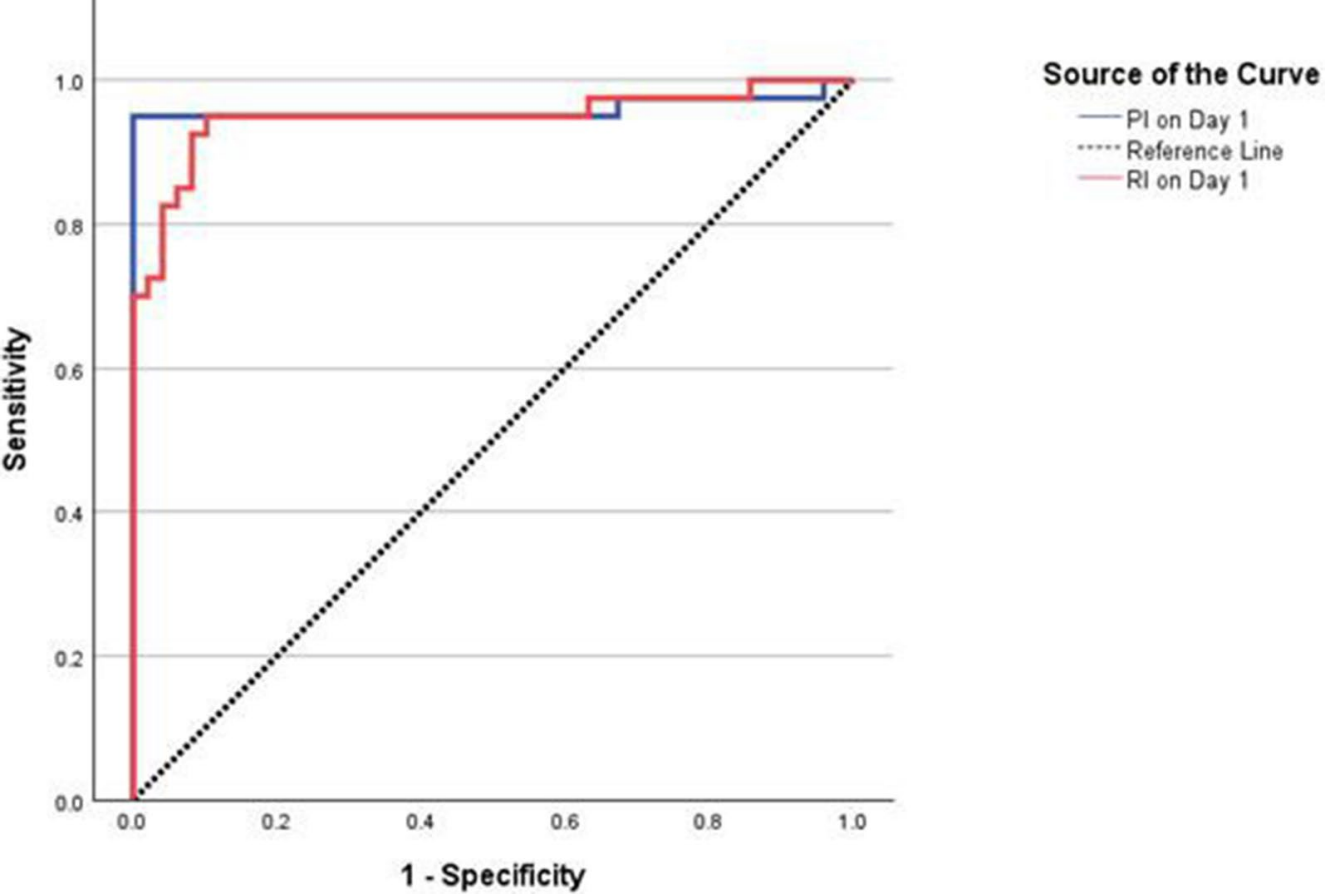
ROC curve of PI and RI on day 1 as predictors of SAE (primary outcome, N = 89). PI showed an AUC of 0.96 (95% CI 0.90–1.01, p < 0.001) at a cut-off ≥ 1.30 (sensitivity 95.0%, specificity 100.0%, accuracy 97.8%, PPV 100.0%, NPV 96.1%). RI showed an AUC of 0.95 (95% CI 0.89–1.00, p < 0.001) at a cut-off ≥ 0.66 (sensitivity 95.0%, specificity 89.8%, accuracy 92.1%, PPV 88.4%, NPV 95.7%). PI, Pulsatility index; *ROC* Receiver Operating Characteristic; *RI* Resistive index

The ROC analysis of the pulsatility and resistive indices for predicting SAE showed that the PI on day 1 had an AUC of 0.96 (95% CI 0.90–1.01, P < 0.001), with 95.0% sensitivity, 100.0% specificity, and 97.8% accuracy at a cut-off ≥ 1.30. Similarly, the RI on day 1 demonstrated an AUC of 0.95 (95% CI 0.89–1.00, P < 0.001), with 95.0% sensitivity, 89.8% specificity, and 92.1% accuracy at a cut-off ≥ 0.66 (Fig. 2, Table 2).

**Table 2:** Accuracy of pulsatility and resistive indices for prediction of SAE (N = 89)

Variable	AUC (95% CI)	Cut-off Point	P value	Sensitivity (%)	Specificity (%)	Accuracy (%)	PPV (%)	NPV (%)
PI on Day 1	0.96 (0.90–1.02)	≥ 1.30	< 0.001*	95.00%	100%	97.75%	100.00%	96.08%
RI on Day 1	0.95 (0.89–1.00)	≥ 0.66	< 0.001*	95.00%	89.80%	92.13%	88.37%	95.65%

*AUC, *Area Under the Curve,* CI, Confidence Interval, NPV *Negative Predictive Value*; PI *Pulsatility Index*, PPV *Positive Predictive Value*, RI *Resistive Index,* SAE *Sepsis-Associated Encephalopathy*. *P values were calculated using the receiver operating characteristic (ROC) curve analysis. * Statistically significant at P < 0.05

### Correlation of PI and RI with clinical parameters

On day 1, PI showed a strong positive correlation with RI (*r* = 0.956, *p* < 0.001), a moderate positive correlation with the SOFA score (*r* = 0.483, *p* < 0.001) and APACHE II score (*r* = 0.503, *p* < 0.001), and a weak positive correlation with age (*r* = 0.230, *p* < 0.05). Conversely, PI demonstrated a strong negative correlation with FVd (*r* = − 0.678, *p* < 0.001) and MAP after resuscitation (*r* = − 0.618, *p* < 0.001), a moderate negative correlation with FVm (*r* = − 0.584, *p* < 0.001), and weak negative correlations with FVs (*r* = − 0.213, *p* < 0.05) and SpO₂ (*r* = − 0.247, *p* < 0.05).

Similarly, RI on day 1 showed moderate positive correlations with the SOFA score (*r* = 0.475, *p* < 0.001) and APACHE II score (*r* = 0.489, *p* < 0.001), as well as a weak positive correlation with age (*r* = 0.265, *p* < 0.05). It also demonstrated strong negative correlations with FVd (*r* = − 0.737, *p* < 0.001) and MAP after resuscitation (*r* = − 0.613, *p* < 0.001), a moderate negative correlation with FVm (*r* = − 0.583, *p* < 0.01), and weak negative correlations with FVs (*r* = − 0.259, *p* < 0.05) and SpO₂ (*r* = − 0.217, *p* < 0.05). The results of the correlation analysis are shown in Supplementary Fig. 2.

### Factors associated with mortality in the SAE group.

In the SAE subgroup, non-survivors showed more severe clinical profiles compared to survivors. Key differences included higher illness severity scores and greater hemodynamic instability. Detailed comparisons of clinical, physiological, and laboratory parameters between survivors and non-survivors. (Supplementary Table 3).

### Cerebral hemodynamic indices in survivors and non-survivors

Cerebral hemodynamic findings comparing survivors and non-survivors within the SAE group are summarized in Supplementary Table 4.

### Predictive performance of PI and RI for mortality

**Fig. 3:**
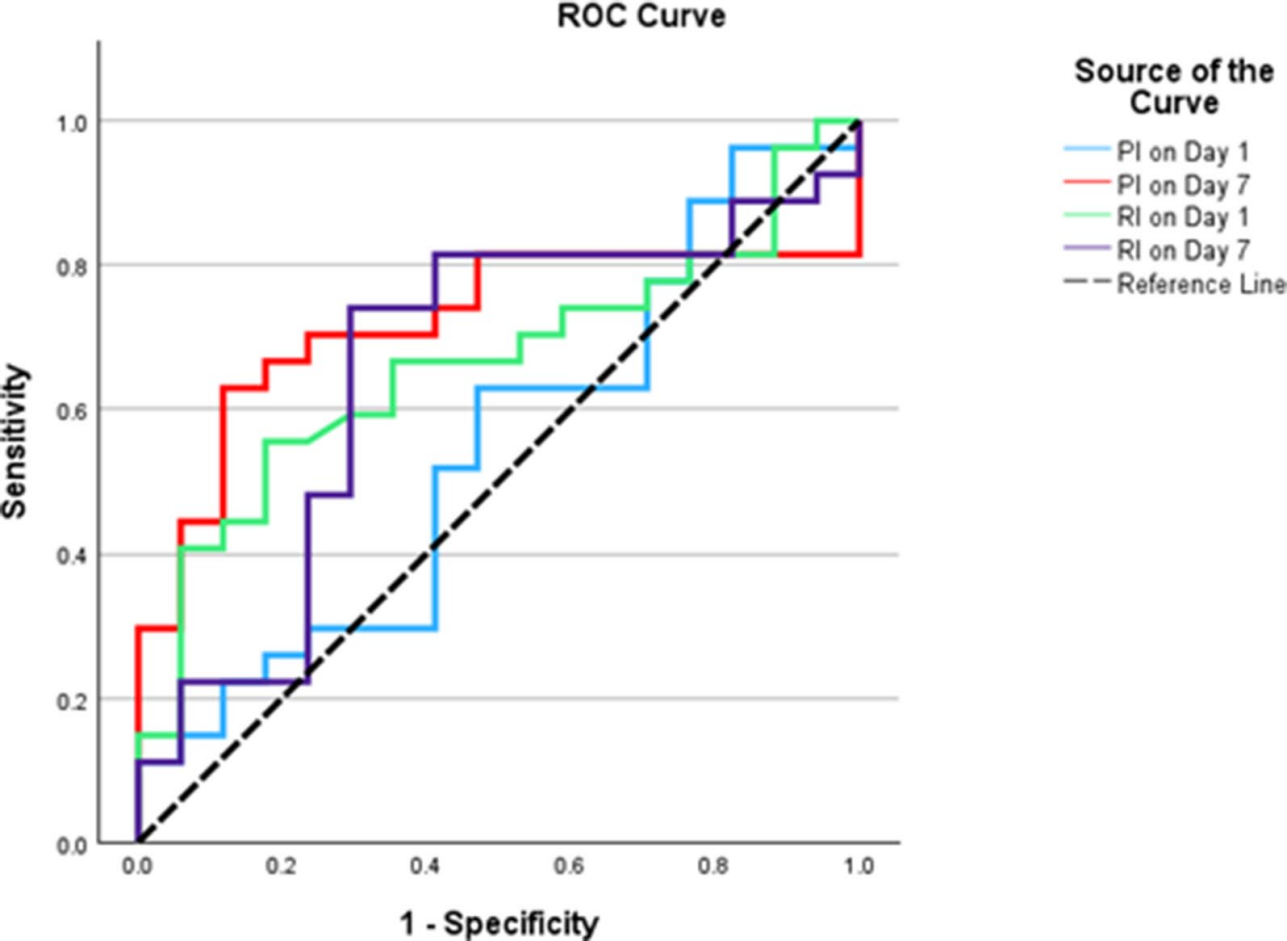
ROC curve of PI and RI as predictors of mortality in the SAE group (secondary outcome, N = 44). PI on day 7 had the highest predictive value (AUC 0.72, 95% CI 0.56–0.88, P = 0.006) with sensitivity 70.4% and specificity 76.5%. RI on day 1 showed a lower AUC (0.34, 95% CI 0.18–0.50, P = 0.047), indicating limited predictive accuracy. *PI* Pulsatility index, *ROC* Receiver Operating Characteristic; *RI* Resistive index

ROC curve analysis revealed that the PI on day 7 had the highest predictive accuracy for mortality in the SAE group, with a significant AUC of 0.72 (95% CI 0.56–0.88, *p* = 0.006) at a cutoff of ≥ 1.50. This resulted in 70.37% sensitivity and 76.47% specificity. Conversely, the RI on day 1 was statistically significant (*AUC* = 0.34, *p* = 0.047) but demonstrated poor predictive performance, with 85.19% sensitivity and only 5.88% specificity (Supplementary Table 5, Fig. 3).

## Discussion

SAE is one of the most common forms of encephalopathy in surgical ICUs and is associated with poor clinical outcomes [14, 15]. However, its diagnosis is often delayed and challenging, primarily due to the lack of specific diagnostic criteria, as diagnosis relies largely on the exclusion of other brain injuries [16, 17]. This creates a critical need for a simple, noninvasive method with high sensitivity and specificity to predict and diagnose SAE early. TCD provides valuable insights into cerebral hemodynamics by measuring blood flow velocities and indices such as PI and RI, which are related to vascular resistance and perfusion in the brain [7].

In this study, we aimed to evaluate the role of TCD as an objective screening tool for predicting the incidence and prognosis of SAE in septic patients. Our results consistently showed higher PI and RI values in SAE patients across all study days. Specifically, PI ranged from 1.51 to 1.58 in SAE patients compared to 0.95 to 1.12 in non-SAE patients, while RI ranged from 0.66 to 0.75 in SAE patients versus 0.56 to 0.64 in non-SAE patients (all p < 0.001). These findings suggest increased vascular resistance and impaired cerebral perfusion, which may play a significant role in the pathophysiology of SAE [4, 18].

Consistent with our findings, Algebaly et al. [8] reported significantly higher PI (1.15 [0.98–1.48] vs. 1.0 [0.95–1.06]) and RI (0.68 [0.61–0.77] vs. 0.62 [0.59–0.64]) in SAE children admitted to the PICU. Similarly, Crippa et al. [4] found a significant association between sepsis-associated brain dysfunction and altered cerebral autoregulation detected by TCD. Furthermore, Schramm et al. [19] demonstrated that cerebrovascular dysregulation on ICU Day 1 was significantly associated with the development of sepsis-associated delirium, further supporting TCD's role in the early prediction of sepsis-related neurological complications. Similarly, multiple studies have linked increased cerebrovascular resistance (CVR) to a higher incidence of delirium and impaired consciousness [20, 21].

In addition to elevated PI and RI, SAE patients exhibited significantly lower mean velocity compared to non-SAE patients, reinforcing the notion that reduced cerebral blood flow serves as a hallmark of SAE [22]. These findings underscore the importance of TCD in identifying subtle changes in cerebral perfusion that may not be apparent through clinical assessment alone.

A significant correlation was observed between TCD indices (PI and RI) and key clinical parameters, including APACHE II, SOFA, and MAP. Specifically, PI values on day 1 demonstrated a strong positive correlation with SOFA and APACHE II scores, suggesting that TCD may serve as a reliable indicator of sepsis severity and systemic organ dysfunction, including neurological impairment [8]. Furthermore, a strong negative correlation between PI/RI and MAP suggests that impaired cerebral blood flow regulation, potentially due to microcirculatory dysfunction, plays a crucial role in the pathogenesis of SAE [23].

ROC analysis of PI and RI showed strong predictive accuracy for SAE over the seven days. Specifically, PI measured on day 1 had the highest accuracy for predicting SAE, with a cutoff value of ≥ 1.30 (95.45% sensitivity, 100% specificity). These findings demonstrate that PI can reliably identify nearly all patients who will develop SAE while excluding those who will not, establishing it as a robust early diagnostic marker. This finding is consistent with Algebaly et al. [8], who reported a PI cutoff of 1.0, with a sensitivity of 73.3% and specificity of 43.3% (AUC = 0.72, *p* = 0.002), supporting its clinical utility in guiding resuscitation and monitoring of SAE patients. However, Zidan et al. [24] classified PI as only a fair predictor of SAE in septic patients. Variations in cutoff values and predictive accuracy may stem from differences in study populations, sepsis severity, or methodological approaches.

On the other hand, RI measured on day 3 demonstrated the highest predictive accuracy (AUC = 0.971, p < 0.001), with a cutoff of ≥ 0.67, achieving 95.45% sensitivity and 95.92% specificity. Consistent with our findings, Algebaly et al. [8] reported that an RI cutoff of 0.62 yielded a sensitivity of 73.3% and specificity of 50.0% (AUC = 0.73, p = 0.001), while Zidan et al. [24] found an RI cutoff of 0.6 with a sensitivity of 72.4% and specificity of 74.1% (AUC = 0.747). Overall, these findings highlight the potential value of early TCD measures in identifying patients at risk of SAE. The high sensitivity and specificity of PI and RI, especially in the early days of ICU admission, suggest that routine TCD monitoring could be integrated into sepsis management protocols to identify patients at risk of neurological deterioration, thereby guiding timely interventions and potentially improving outcomes.

The study additionally examined the use of TCD parameters to predict mortality among SAE patients. Non-survivors exhibited progressive cerebral hemodynamic deterioration, characterized by higher PI and lower mean velocity from day 5 to day 7. Algebaly et al. [8] also reported higher PI and RI values in pediatric SAE non-survivors. This may be explained by the fact that PI may be more sensitive than RI in detecting changes in cerebrovascular resistance (CVR) [25, 26]. Other studies have shown similar links between higher CVR and increased mortality [27, 28].

ROC curve analysis identified PI on day 7 as the most accurate predictor of mortality in SAE patients (AUC = 0.719, *p* = 0.006) at a cutoff of ≥ 1.50, with 70.37% sensitivity and 76.47% specificity. In contrast, RI on day 1, despite being statistically significant (AUC = 0.337, *p* = 0.047), demonstrated poor predictive performance (85.19% sensitivity, 5.88% specificity). This aligns with Algebaly et al. [8], who reported a stronger predictive role for both PI and RI in assessing pediatric SAE mortality.

The incidence of SAE in our study was 47.3% (n = 44). SAE patients were significantly older, which aligns with previous studies suggesting that age increases susceptibility to SAE [15, 28].

A higher incidence of septic shock on day 1 was observed in SAE patients (84.1% vs. 63.3%, *p* = 0.024), indicating greater initial disease severity in this group. This was further supported by significantly higher SOFA scores on both day 1 (6 vs. 5, *p* < 0.001) and day 7 (8 vs. 5, *p* < 0.001), as well as higher APACHE II scores (14 vs. 11, *p* < 0.001), suggesting a higher overall severity of illness and a greater risk of mortality. Both SOFA and APACHE II scores have been shown to accurately predict hospital mortality rates and median survival time in sepsis patients [29].

Although SAE patients had a longer ICU stay, the difference did not reach statistical significance (*p* = 0.086). This suggests that while SAE may not always prolong ICU admission, it significantly impairs clinical outcomes [3].

However, it is important to recognize that PI and RI may partially reflect the overall severity of systemic illness and should therefore be interpreted in the context of the patient’s global clinical status rather than as completely independent predictors. This perspective provides a balanced interpretation of our results and emphasizes that PI and RI should be considered alongside other clinical and laboratory parameters when assessing patient prognosis.

While the use of TCD ultrasonography offers a promising non-invasive method to assess cerebral perfusion in septic patients, its implementation in routine ICU practice is not without challenges. TCD requires trained and experienced operators to ensure reliable insonation of cerebral arteries and reproducibility of Doppler measurements. Interobserver variability, especially in obtaining accurate angle alignment and identifying optimal flow windows, remains a known limitation that could affect consistency across centers. Moreover, in resource-limited settings, the availability of TCD equipment and skilled personnel may hinder daily application. Despite these obstacles, TCD remains more accessible and affordable compared to advanced neuromonitoring modalities such as continuous EEG or cerebral microdialysis. With appropriate training programs and protocol standardization, bedside TCD could be feasibly integrated into clinical practice, offering a practical tool for early detection of sepsis-associated cerebral dysfunction.

## Strengths and limitations

To our knowledge, this is the first prospective study to systematically monitor TCD parameters over seven consecutive days in adult septic patients, offering a longitudinal view of cerebral hemodynamic changes associated with SAE. Unlike previous studies that evaluated TCD parameters at isolated time points or in pediatric populations, our study provides a comprehensive temporal profile that may enhance early diagnosis and risk stratification. All TCD assessments were performed by a single experienced operator, minimizing inter-rater variability and enhancing the reliability and consistency of the measurements. The study also identifies PI and RI as potential early predictors for SAE and mortality, with ROC analysis demonstrating their predictive accuracy.

This study has several limitations. First, it was conducted at a single center (Menoufia University Hospitals), which may limit generalizability. Second, although powered to assess the AUROC of RI on Day 1, analyses across multiple timepoints and indices were exploratory. Third, long-term neurological outcomes were not evaluated, and deeply sedated or comatose patients (RASS ≤ –2) were excluded, potentially underrepresenting severe SAE cases. The absence of EEG or advanced neurophysiological monitoring also limited accurate assessment of encephalopathy. Finally, we were unable to examine dose–response relationships between worsening PI/RI values and SAE severity or outcomes. To enhance the clinical application of TCD in septic patients, future studies should include larger, multicenter cohorts, incorporate neuroimaging for validation, and assess long-term cognitive outcomes. Investigating interventions to modify cerebral perfusion in SAE patients could also provide valuable insights into improving prognosis.

## Conclusions

This study underscores the prognostic value of TCD in SAE, demonstrating that cerebral hemodynamic indices, particularly PI and RI, are significantly altered in SAE patients. PI on day 1 and RI on day 3 exhibited high predictive accuracy for SAE diagnosis. These findings suggest that TCD may serve as a simple and noninvasive tool for the early identification of SAE and risk stratification in septic patients.

## Supplementary Information

Supplementary file 1. Figure 1: Flowchart of the study

Supplementary file 2. Figure 2: Spearman correlation matrix between PI and RI and other clinical data in SAE patients (exploratory analysis, N=44). APACHE II, Acute Physiology and Chronic Health Evaluation II; FVm, mean velocity; FVd, end-diastolic velocity; FVs, peak systolic velocity; MAP, Mean Arterial Pressure; PI, Pulsatility Index; RI, Resistive Index; SOFA, Sequential Organ Failure Assessment; SpO₂, Peripheral Oxygen Saturation.

Supplementary file 3.

## Data Availability

Data is available upon reasonable request from the corresponding author.
