# Supplementary figures and images for: The role of transcranial Doppler in predicting the incidence and prognosis of sepsis-associated encephalopathy
Source: Intensive Care Med Exp. 2025 Dec 15;13:129. doi: 10.1186/s40635-025-00826-9 (PMC12705473; doi:10.1186/s40635-025-00826-9)

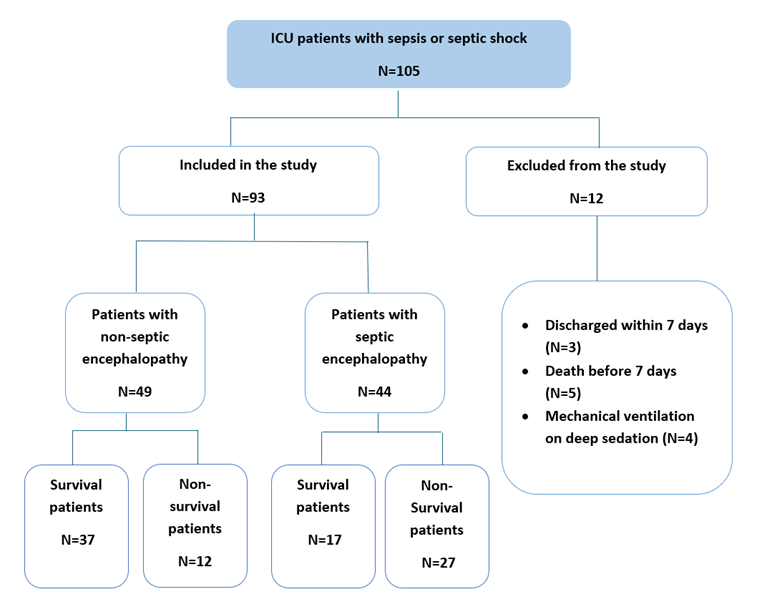

Supplement: Supplementary file 1 — Figure 1: Flowchart of the study [file 40635_2025_826_MOESM1_ESM.tif]

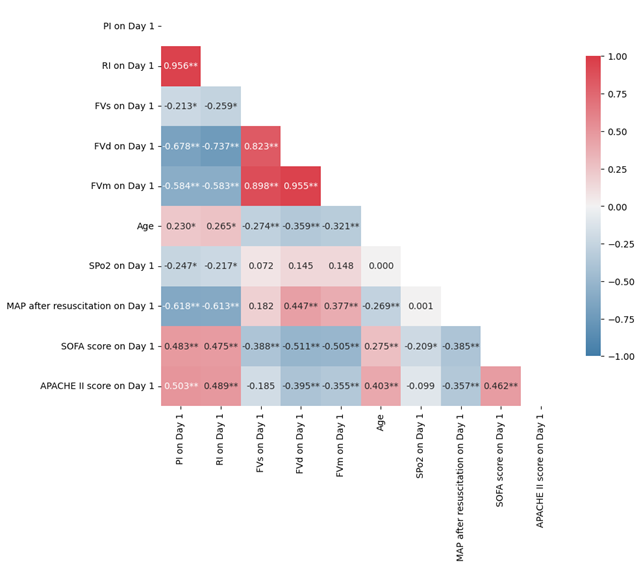

Supplement: Supplementary file 2 — Figure 2: Spearman correlation matrix between PI and RI and other clinical data in SAE patients (exploratory analysis, N=44). APACHE II, Acute Physiology and Chronic Health Evaluation II; FVm, mean velocity; FVd, end-diastolic velocity; FVs, peak systolic velocity; MAP, Mean Arterial Pressure; PI, Pulsatility Index; RI, Resistive Index; SOFA, Sequential Organ Failure Assessment; SpO₂, Peripheral Oxygen Saturation. [file 40635_2025_826_MOESM2_ESM.tif]
