# Supplementary material for: The role of transcranial Doppler in predicting the incidence and prognosis of sepsis-associated encephalopathy
Source: Intensive Care Med Exp. 2025 Dec 15;13:129. doi: 10.1186/s40635-025-00826-9 (PMC12705473; doi:10.1186/s40635-025-00826-9)
Supplement: Supplementary file 3 [file 40635_2025_826_MOESM3_ESM.docx]

**Table 1: Hemodynamic and laboratory parameters of the studied groups (N=93).**

| **Variable** | **SAE patients (n=44)** | **Non-SAE patients (n=49)** | **P-value** |
| --- | --- | --- | --- |
| **SPO₂ on Day 1 (on oxygen, %)**, median (IQR) | 89 (88–93) | 91 (89–95) | 0.122 |
| **SPO2 on Day 7 (%)**, median (IQR) | 93 (89–95) | 94 (91–96) | 0.286 |
| **MAP after resuscitation on Day 1 (mmHg)**, median (IQR) | 73 (70–75) | 79 (77–81) | **<0.001*** |
| **MAP on Day 7 (mmHg)**, median (IQR) | 82 (78–89) | 84 (81–87) | 0.112 |
| **Temperature (°C) on Day 1 (bpm)**, median (IQR) | 38.1 (37.9–39.0) | 38.3 (38.0–38.9) | 0.360 |
| **Temperature (°C) on Day 7 (bpm)**, median (IQR) | 38.2 (37.8–38.7) | 37.5 (37.3–37.6) | **<0.001*** |
| **Heart rate on Day 1 (bpm)**, mean ± SD | 117 ± 13 | 112 ± 11 | **0.030*** |
| **Heart rate on Day 7 (bpm)**, mean ± SD | 105 ± 14 | 93 ± 16 | **<0.001*** |
| **Hemoglobin (g/dL)**, median (IQR) | 9.0 (8.4–10.0) | 9.3 (9.0–10.0) | 0.238 |
| **TLC (×10⁹/L)**, median (IQR) | 19.9 (15.5–25.0) | 19.0 (15.3–22.0) | 0.361 |
| **CRP (mg/L)**, median (IQR) | 94.0 (81.0–108.5) | 89.0 (80.0–98.0) | 0.205 |
| **Procalcitonin (ng/mL)** , median (IQR) | 6.00 (1.85–11.50) | 2.00 (1.50–2.50) | **<0.001*** |
| **Creatinine (mg/dL)**, median (IQR) | 1.5 (1.2–2.1) | 1.3 (1.0–1.9) | 0.166 |
| **Urea (mg/dL)**, median (IQR) | 45 (39–72) | 40 (19–50) | **0.019*** |
| **ALT (U/L)**, median (IQR) | 22 (19–29) | 20 (15–30) | 0.331 |
| **Sodium (mEq/L)**, median (IQR) | 138 (135–140) | 137 (135–140) | 0.756 |
| **Potassium (mEq/L)**, median (IQR) | 4.0 (4.0–4.3) | 4.0 (3.9–4.2) | 0.546 |
| **Ionized Calcium (mmol/L)**, median (IQR) | 1.10 (1.00–1.20) | 1.10 (1.00–1.21) | 0.103 |
| **Magnesium (mg/dL)**, median (IQR) | 1.90 (1.80–2.00) | 1.90 (1.80–2.10) | 0.747 |

*ALT, Alanine Aminotransferase; bpm, Beats Per Minute; CRP, C-Reactive Protein; MAP, Mean Arterial Pressure; SAE, Sepsis-Associated Encephalopathy; SpO₂, Peripheral Oxygen Saturation; TLC, Total Leukocyte Count.*

*Data presented as median (interquartile range) or mean ± standard deviation. P values for independent samples t-test, or Mann-Whitney U test, as appropriate, were recorded,* ******Statistically significant at P < 0.05.*

**Table 2: Comparison of cerebral hemodynamic parameters between SAE and non-SAE patients over seven days (N=93).**

| **Parameter** | | **SAE patients (n=44)** | **Non- SAE patients (n=49)** | **P-value** |
| --- | --- | --- | --- | --- |
| **Pulsatility index** | **Day 1** | 1.51 (1.45 – 1.58) | 1.02 (0.94 – 1.12) | **<0.001*** |
|  | **Day 2** | 1.53 (1.48 – 1.61) | 1.01 (0.95 – 1.08) | **<0.001*** |
|  | **Day 3** | 1.56 (1.44 – 1.64) | 1.01 (0.93 – 1.06) | **<0.001*** |
|  | **Day 4** | 1.51 (1.40 – 1.68) | 0.96 (0.93 – 1.06) | **<0.001*** |
|  | **Day 5** | 1.52 (1.36 – 1.71) | 0.95 (0.91 – 1.04) | **<0.001*** |
|  | **Day 6** | 1.54 (1.34 – 1.78) | 0.93 (0.90 – 1.00) | **<0.001*** |
|  | **Day 7** | 1.58 (1.27 – 1.81) | 0.95 (0.90 – 0.99) | **<0.001*** |
| **Resistive index** | **Day 1** | 0.72 (0.70 – 0.74) | 0.60 (0.57 – 0.64) | **<0.001*** |
|  | **Day 2** | 0.73 (0.70 – 0.75) | 0.60 (0.58 – 0.63) | **<0.001*** |
|  | **Day 3** | 0.73 (0.71 – 0.74) | 0.59 (0.57 – 0.63) | **<0.001*** |
|  | **Day 4** | 0.72 (0.71 – 0.75) | 0.58 (0.57 – 0.62) | **<0.001*** |
|  | **Day 5** | 0.72 (0.69 – 0.74) | 0.58 (0.56 – 0.61) | **<0.001*** |
|  | **Day 6** | 0.72 (0.67 – 0.74) | 0.57 (0.56 – 0.59) | **<0.001*** |
|  | **Day 7** | 0.72 (0.66 – 0.75) | 0.57 (0.56 – 0.60) | **<0.001*** |
| **Mean velocity** | **Day 1** | 47.25 (42.38 – 54.65) | 70.50 (54.50 – 77.00) | **<0.001*** |
|  | **Day 2** | 44.00 (41.33 – 51.25) | 70.50 (58.00 – 77.00) | **<0.001*** |
|  | **Day 3** | 42.00 (40.83 – 47.00) | 70.50 (62.00 – 75.50) | **<0.001*** |
|  | **Day 4** | 42.00 (39.75 – 46.20) | 70.00 (63.00 – 74.50) | **<0.001*** |
|  | **Day 5** | 41.78 (37.75 – 46.75) | 70.50 (65.50 – 73.50) | **<0.001*** |
|  | **Day 6** | 40.63 (35.00 – 50.10) | 71.50 (66.50 – 75.00) | **<0.001*** |
|  | **Day 7** | 39.95 (32.25 – 50.50) | 72.00 (68.00 – 74.50) | **<0.001*** |

*SAE, Sepsis-Associated Encephalopathy. Data presented as median (interquartile range). P values for the Mann-Whitney U test.* ******* *Statistically significant at P < 0.05*

**Table 3: Baseline characteristics, comorbidities, sources of sepsis, scores, outcomes, hemodynamics, and laboratory parameters of survivors and non-survivors (N=44).**

| **Variable** | **Survivors (n=17)** | **Non-Survivors (n=27)** | **P-value** |
| --- | --- | --- | --- |
| **Gender**, n (%) |  |  | 0.122 |
| - Female | 6 (35.3%) | 16 (59.3%) |  |
| - Male | 11 (64.7%) | 11 (40.7%) |  |
| **Age**, median (IQR) | 55 (49–58) | 57 (53–58) | 0.845 |
| **BMI**, median (IQR) | 23 (22–25) | 23 (21–30) | 0.559 |
| **DM**, n (%) | 13 (76.5%) | 21 (77.8%) | >0.999 |
| **HTN**, n (%) | 10 (58.8%) | 21 (77.8%) | 0.180 |
| **CKD**, n (%) | 5 (29.4%) | 10 (37.0%) | 0.603 |
| **oncological disease**, n (%) | 7 (41.2%) | 13 (48.1%) | 0.651 |
| **Compensated chronic liver disease**, n (%) | 8 (47.1%) | 10 (37.0%) | 0.510 |
| **Cardiac disease**, n (%) | 11 (64.7%) | 22 (81.5%) | 0.289 |
| **Obstructive lung disease**, n (%) | 6 (35.3%) | 11 (40.7%) | 0.718 |
| **Pulmonary embolism**, n (%) | 0 (0.0%) | 5 (18.5%) | 0.139 |
| **DVT**, n (%) | 5 (29.4%) | 12 (44.4%) | 0.319 |
| **Pneumonia on admission**, n (%) | 12 (70.6%) | 21 (77.8%) | 0.724 |
| **Uro-sepsis**, n (%) | 12 (70.6%) | 15 (55.6%) | 0.319 |
| **Abdominal sepsis**, n (%) | 3 (17.6%) | 13 (48.1%) | **0.041*** |
| **Mechanical ventilation**, n (%) | 3 (17.6%) | 14 (51.9%) | **0.023*** |
| **Septic shock on day 1**, n (%) | 16 (94.1%) | 21 (77.8%) | 0.220 |
| **Septic shock on day 7**, n (%) | 6 (35.3%) | 17 (63.0%) | 0.074 |
| **SOFA score on Day 1**, median (IQR) | 6 (6–7) | 6 (5–7) | 0.941 |
| **SOFA score on Day 7**, mean ± SD | 5.41 ± 1.33 | 8.89 ± 2.64 | **<0.001*** |
| **APACHE II score**, mean ± SD | 13.76 ± 3.01 | 15.04 ± 3.13 | 0.190 |
| **ICU Length of Stay (days)**, mean ± SD | 20.35 ± 7.00 | 18.96 ± 6.71 | 0.514 |
| **SpO₂ on day 1**, median (IQR) | 90 (88–95) | 89 (88–91) | 0.138 |
| **SpO₂ on day 7**, mean ± SD | 94.24 ± 4.12 | 91.30 ± 2.91 | **0.008*** |
| **Mean arterial blood pressure after resuscitation on day 1**, mean ± SD | 74.29 ± 3.00 | 71.67 ± 3.52 | **0.015*** |
| **Mean arterial blood pressure on day 7**, median (IQR) | 85 (79–88) | 80 (77–89) | 0.246 |
| **Temperature °C on day 1**, median (IQR) | 38.9 (37.9–39.6) | 38.1 (37.9–38.5) | 0.224 |
| **Temperature on day 7**, mean ± SD | 37.89 ± 0.54 | 38.40 ± 0.49 | **0.002*** |
| **Heart rate on day 1**, mean ± SD | 118.24 ± 15.19 | 116.96 ± 12.51 | 0.764 |
| **Heart rate on day 7**, mean ± SD | 96.71 ± 14.64 | 109.48 ± 11.99 | **0.003*** |
| **Hemoglobin**, median (IQR) | 9.50 (9.00–10.70) | 9.00 (8.00–9.30) | **0.017*** |
| **TLC**, mean ± SD | 18.50 ± 7.89 | 21.37 ± 6.60 | 0.200 |
| **CRP**, median (IQR) | 90.0 (76.0–103.0) | 97.0 (81.0–110.0) | 0.347 |
| **Procalcitonin**, median (IQR) | 5.00 (1.80–12.00) | 7.00 (2.20–11.00) | 0.392 |
| **Creatinine**, median (IQR) | 1.3 (1.0–2.0) | 1.5 (1.2–2.3) | 0.240 |
| **Urea**, median (IQR) | 45 (39–78) | 45 (39–70) | 0.904 |
| **ALT**, median (IQR) | 29 (20–40) | 20 (19–29) | **0.034*** |
| **Sodium**, mean ± SD | 137.76 ± 3.68 | 137.33 ± 2.67 | 0.655 |
| **Potassium**, median (IQR) | 4.0 (3.9–4.2) | 4.0 (4.0–4.3) | 0.504 |
| **Ionized Calcium**, median (IQR) | 1.10 (1.00–1.10) | 1.10 (1.00–1.20) | 0.467 |
| **Magnesium**, median (IQR) | 1.90 (1.80–2.00) | 1.80 (1.80–2.10) | 0.696 |

*ALT, Alanine Aminotransferase; APACHE II, Acute Physiology and Chronic Health Evaluation II; BMI, Body Mass Index; CAM-ICU, Confusion Assessment Method for the ICU; CKD, Chronic Kidney Disease; CRP, C-Reactive Protein; DM, Diabetes Mellitus; DVT, Deep Vein Thrombosis; HTN, Hypertension; ICU, Intensive Care Unit;* *SAE, Sepsis-Associated Encephalopathy; SOFA, Sequential Organ Failure Assessment; SpO₂, Oxygen Saturation; TLC, Total Leukocyte Count.*

*Data presented as frequency (percentage) for categorical variables, median (interquartile range), or mean ± standard deviation for continuous variables. P values were calculated using a chi-square test for categorical variables, a Mann-Whitney U test, or an independent samples t-test for continuous variables.* ***** *Statistically significant at P<0.05.*

**Table 4: Comparison of cerebral hemodynamic parameters between survivors and non-survivors in the SAE group over seven days (N=44).**

| **Parameter** | | **Survivors (n=17)** | **Non-Survivors**  **(n=27)** | **P-value** |
| --- | --- | --- | --- | --- |
| **Pulsatility index** | **Day 1** | 1.52 (1.47–1.58) | 1.51 (1.43–1.57) | 0.638 |
|  | **Day 2** | 1.52 (1.47–1.61) | 1.55 (1.48–1.64) | 0.462 |
|  | **Day 3** | 1.49 (1.44–1.61) | 1.60 (1.44–1.72) | 0.219 |
|  | **Day 4** | 1.47 (1.40–1.56) | 1.60 (1.40–1.72) | 0.159 |
|  | **Day 5** | 1.40 (1.34–1.51) | 1.62 (1.42–1.73) | **0.044*** |
|  | **Day 6** | 1.40 (1.32–1.45) | 1.72 (1.38–1.82) | **0.033*** |
|  | **Day 7** | 1.33 (1.25–1.48) | 1.75 (1.34–1.86) | **0.015*** |
| **Resistive index** | **Day 1** | 0.73 (0.72–0.74) | 0.71 (0.69–0.74) | 0.071 |
|  | **Day 2** | 0.73 (0.73–0.74) | 0.72 (0.69–0.75) | 0.173 |
|  | **Day 3** | 0.73 (0.71–0.74) | 0.73 (0.71–0.75) | 0.895 |
|  | **Day 4** | 0.73 (0.71–0.74)- | 0.72 (0.70–0.75) | 0.782 |
|  | **Day 5** | 0.71 (0.68–0.72) | 0.72 (0.70–0.74) | 0.379 |
|  | **Day 6** | 0.68 (0.66–0.72) | 0.73 (0.71–0.75) | 0.132 |
|  | **Day 7** | 0.68 (0.65–0.74) | 0.74 (0.69–0.75) | 0.085 |
| **Mean velocity** | **Day 1** | 45.00 (41.50–48.00) | 47.50 (43.00–54.80) | 0.205 |
|  | **Day 2** | 43.00 (41.00–45.50) | 44.25 (41.70–51.50) | 0.613 |
|  | **Day 3** | 42.10 (41.50–44.35) | 41.75 (40.05–47.00) | 0.726 |
|  | **Day 4** | 45.00 (41.00–47.50) | 41.15 (38.50–44.00) | 0.080 |
|  | **Day 5** | 46.50 (44.50–48.00) | 39.00 (36.00–42.50) | **<0.001*** |
|  | **Day 6** | 50.00 (43.30–51.50) | 35.50 (33.00–41.25) | **<0.001*** |
|  | **Day 7** | 49.35 (42.50–56.00) | 32.80 (31.50–40.50) | **<0.001*** |

*SAE, Sepsis-Associated Encephalopathy. Data presented as median (interquartile range). P values for the Mann-Whitney U test.* ******* *Statistically significant at P<0.05.*

**Table 5: Accuracy of PI and RI in prediction of mortality in the SAE group (N=44).**

| **Variable** | **AUC (95% CI)** | **Cut-Off Point** | **P value** | **Sensitivity**  **(%)** | **Specificity**  **(%)** | **Accuracy**  **(%)** | **PPV**  **(%)** | **NPV**  **(%)** |
| --- | --- | --- | --- | --- | --- | --- | --- | --- |
| **PI on Day 1** | 0.46 (0.28–0.64) | ≥ 1.50 | 0.640 | 70.37% | 41.18% | 59.09% | 65.52% | 46.67% |
| **PI on Day 7** | 0.72 (0.56–0.88) | ≥ 1.50 | **0.006*** | 70.37% | 76.47% | 72.73% | 82.61% | 61.90% |
| **RI on Day 1** | 0.34 (0.18–0.50) | ≥ 0.68 | **0.047*** | 85.19% | 5.88% | 54.55% | 58.97% | 20.00% |
| **RI on Day 7** | 0.66 (0.48–0.83) | ≥ 0.69 | 0.077 | 81.48% | 58.82% | 72.73% | 75.86% | 66.67% |

*AUC, Area Under the Curve; CI, Confidence Interval; NPV, Negative Predictive Value; PI, Pulsatility Index; PPV, Positive Predictive Value; RI, Resistive Index;* *SAE, Sepsis-Associated Encephalopathy. P values were calculated using the receiver operating characteristic (ROC) curve analysis.* ***** *Statistically significant at P<0.05.*
